# Interactions between *Babesia microti* merozoites and rat kidney cells in a short-term in vitro culture and animal model

**DOI:** 10.1038/s41598-021-03079-0

**Published:** 2021-12-08

**Authors:** Marta Albertyńska, Hubert Okła, Krzysztof Jasik, Danuta Urbańska-Jasik, Przemysław Pol

**Affiliations:** 1 Department of Pathology, Faculty of Pharmaceutical Sciences in Sosnowiec, Medical University of Silesia in Katowice, Ostrogórska 30, 41-200 Sosnowiec, Poland; 2 Provincial Sanitary and Epidemiological Station in Katowice, Raciborska 39, 40-074 Katowice, Poland; 3 Faculty of Science and Technology, University of Silesia in Katowice, 75 Pułku Piechoty 1, 41-500 Chorzów, Poland; 4 Institute of Biology, Biotechnology and Environmental Protection, Faculty of Natural Sciences, University of Silesia in Katowice, Bankowa 9, 40-007 Katowice, Poland; 5 Department of Small Livestock Breeding, The National Research Institute of Animal in Kraków, Krakowska 1, 32-083 Balice, Poland

**Keywords:** Parasitology, Mechanisms of disease

## Abstract

Babesiosis is one of the most common infections in free-living animals and is rapidly becoming significant among human zoonoses. Cases of acute renal failure in humans caused by *Babesia* spp. have been described in the literature. The kidneys are characterised by intense blood flow through the blood vessels, which increases the likelihood of contact with the intra-erythrocyte parasite. The aim of this study was to observe the influence of *B. microti* (ATCC 30221) on renal epithelial cells in vitro cultured (NRK-52E line) and Wistar rats’ kidney. Both NRK-52E cells and rats’ kidney sections were analysed by light microscopy, transmission electron microscopy (TEM) and fluorescence in situ hybridization (FISH). Necrotic changes in renal epithelial cells have been observed in vitro and in vivo. In many cross-sections through the rats’ kidney, adhesion of blood cells to the vascular endothelium, accumulation of erythrocytes and emboli were demonstrated. In NRK-52E culture, elements with a distinctly doubled cell membrane resembling *B. microti* were found inside the cytoplasm and adjacent to the cell layer. The study indicates a chemotactic tendency for *B. microti* to adhere to the renal tubules' epithelium, a possibility of piroplasms entering the renal epithelial cells, their proliferation within the cytoplasm and emboli formation.

## Introduction

Babesiosis is a parasitic tick-borne disease caused by apicomplexan, intra-erythrocytic parasites of the genus *Babesia*. Apart from Lyme disease and tick-borne encephalitis, babesiosis is one of the most commonly reported tick-borne diseases globally. Babesiosis, apart from trypanosomosis, theileriosis and anaplasmosis, belongs to the important veterinary hemoprotozoan diseases^1^. Due to the significant increase in the number of infections in recent years, piroplasmosis belongs to emerging tick-borne diseases^2–4^. The observed increase in the incidence of tick-borne diseases in humans, including babesiosis, results from climate and landscape changes, increased interactions between humans and the environment, increased immunosuppression and changes in the abundance of host and vector species^5^. Babesiosis is one of the most common infections in free-living animals and is rapidly becoming significant among human zoonoses^6^.

In most infected people, with a properly working immune system, the disease is asymptomatic or mild (flu-like symptoms). Some patients above 50, especially immunocompromised or with comorbidities, may have exacerbated symptoms and develop a severe, complicated form of babesiosis^7,8^. Asymptomatic carriage can cause horizontal infection when the carrier donates blood, and vertical infection when pregnant^9,10^. The first symptoms of babesiosis appear after an incubation period, which can last from 1 to 4 weeks after a tick bite, or even up to 9 weeks in the case of transmission of the protozoan by blood transfusion or blood products^11,12^. Non-specific general symptoms such as malaise and fatigue, high fever often accompanied by chills and sweating, headache, muscle and joint pain, and eating disorders are characteristic for the initial stage of infection in human. Among less common symptoms include photophobia, nausea, vomiting, stomach and throat pain, cough, as well as hyperalgesia, emotional instability and depression^13,14^. Spleen and liver enlargement, pharyngitis, jaundice, and retinopathy are occasionally found^15^. Laboratory tests have shown haemolytic anemia as manifested by low hemoglobin levels and low hematocrit, with hemoglobinuria, as well as thrombocytopenia and an increase in reticulocytes. The acute phase of babesiosis lasts about 1 to 2 weeks, but fatigue, malaise and anaemia may persist for several months^16^. The exacerbation of symptoms and complications from various organs, such as damage of kidney, liver, spleen, congestive heart failure, acute respiratory failure, intravascular coagulation syndrome or disorders in the central nervous system, depending on the patient's immune system status^17,18^. Babesiosis can be severe and even fatal, especially in immunocompromised patients treated with immunosuppressants after transplantation or in the course of cancer, in patients infected with HIV and after splenectomy. Infection is dangerous for premature babies, the elderly and patients with hemoglobinopathy, chronic heart, lung, or liver disease undergoing anti-cytokine therapy^19–21^.

The kidneys are well supplied with blood and are characterised by intense blood flow through the blood vessels, which increases the likelihood of contact with the intra-erythrocyte parasite. Cases of acute renal failure in humans caused by *Babesia* spp. have been described in the literature^22^.

This study aimed to observe the interactions between *B. microti* and rats’ kidney at the microscopic and submicroscopic level. At the same time, in vitro analysis of the interactions between *B. microti* and kidney cells was performed. An additional aim was to compare protozoa's effect on renal epithelial cells in vivo and in vitro conditions.

## Results

**Figure 1:**
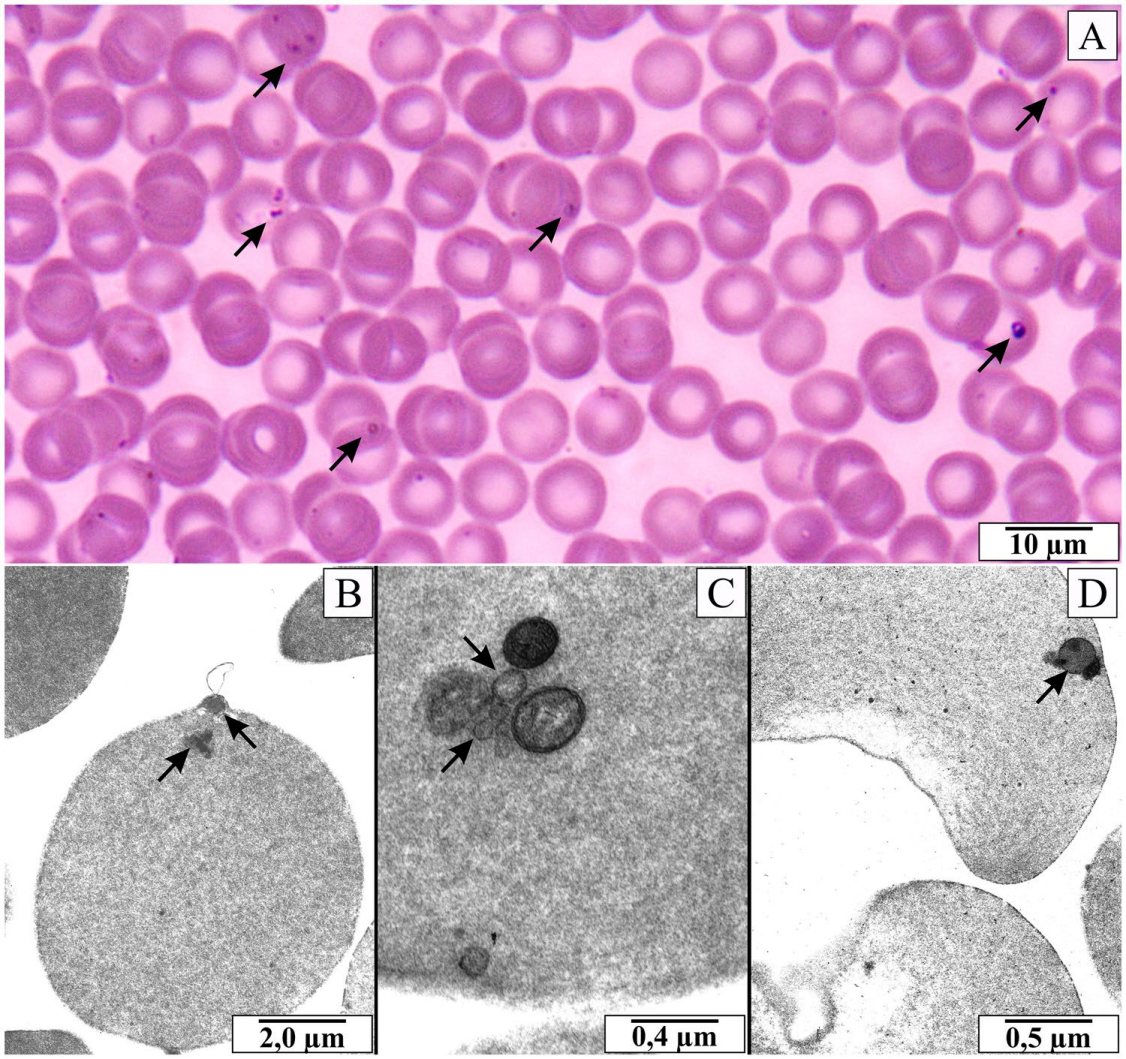
Blood smear of rats infected with *B. microti*. (**A**) blood smear stained by the May-Grünwald-Giemsa method. (**B**–**D**) electronmicrographs of blood cells. Arrows indicate different forms of *B. microti* merozoites inside erythrocytes.

**Figure 2:**
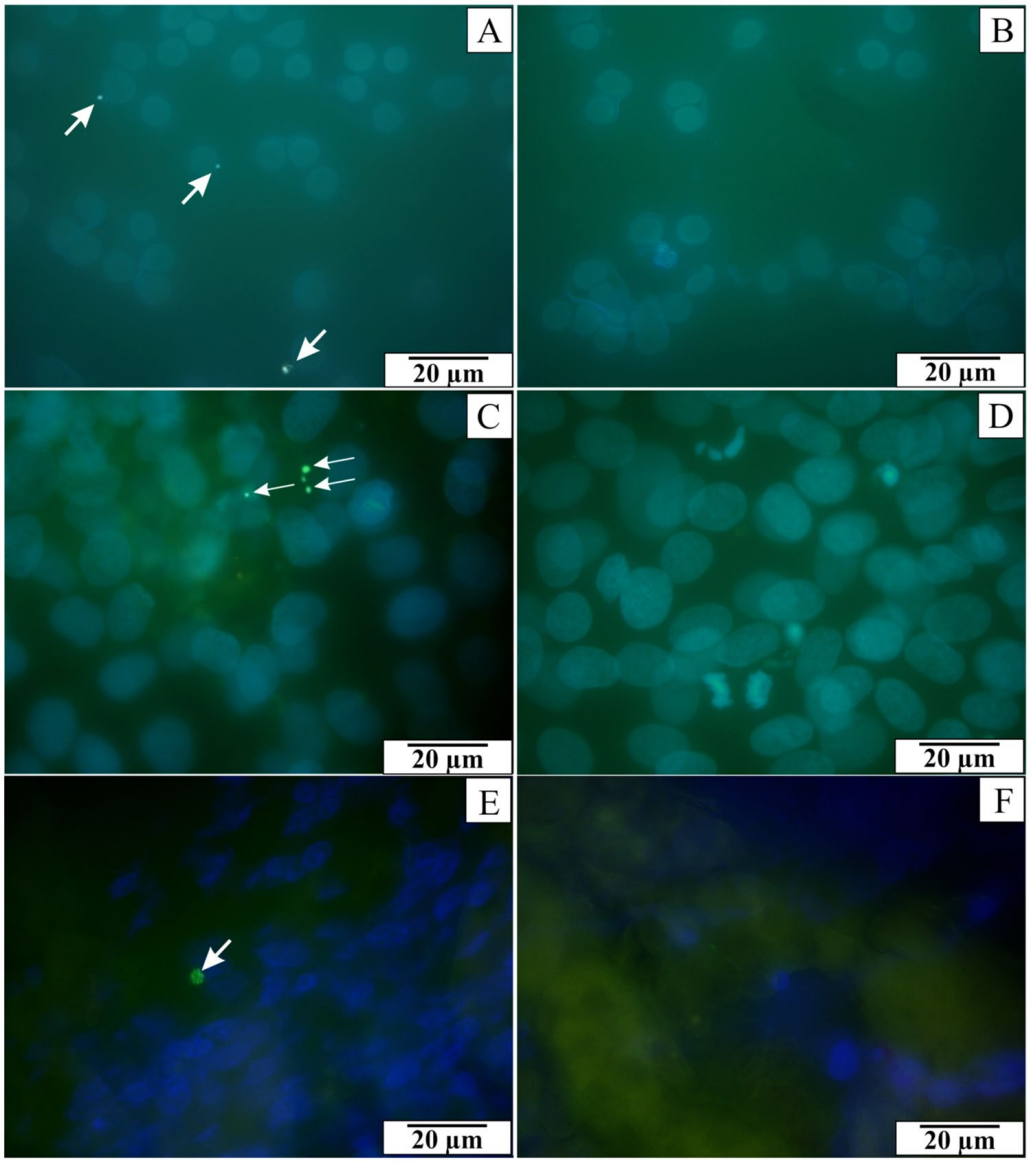
Fluorescent in situ hybridization. (**A**) blood smear of rats infected with *B. microti*. (**B**) blood smear of rats from control group. (**C**) rats’ kidney epithelial cells (NRK-52E cells) cultured with blood collected from infected rats. (**D**) control NRK-52E cells. (**E**) kidney collected from rats infected with *B. microti*. (**F**) kidney collected from control rats. Arrows in figures (**A**,**C**,**E**) indicate green spot signals of fluorescence which confirm the presence of *B. microti* genetic material.

The observations confirmed the presence of *B. microti* in the blood cells of infected rats. The parasitemia of the rats on the 21st day after infection ranged from 15.6 to 27.8% (mean—22.46% ± 3.64%). Various shapes of *B. microti* merozoites (oval, pear-shaped or ring-shaped) and varying numbers of inclusions were observed inside the erythrocytes (Fig. 1A). Additionally, the electronmicrographs show dark, osmophilic inclusions inside the blood cells (Fig. 1B–D). The presence of *B. microti* genetic material in the rats’ blood was confirmed to complement the microscopic diagnosis. Green spot signals of fluorescence were detected when the probe has connected to complementary DNA of *B. microti* (Fig. 2A). Similar green spots were observed inside NRK-52E cells cultured with protozoa (Fig. 2C) and in sections of rats’ kidney on the 21st day of parasitemia (Fig. 2E). Fluorescence signals were present in many fields of observation. No fluorescence signals were detected in blood smears of rats from the control group (Fig. 2B), in preparations from the control in vitro culture of NRK-52E cells (Fig. 2D) and in sections of control rats’ kidney (Fig. 2F). The results confirmed that merozoites could enter cells both in in vitro culture and animal model.

In addition, microscopic analysis of the rats’ blood, which were inoculated with NRK-52E cells, showed the presence of various shapes of *B. microti* merozoites in erythrocytes similar to the rats from the study group.

### Histological and ultrastructural analysis of the effect of *B. microti* on NRK-52E cells

**Figure 3:**
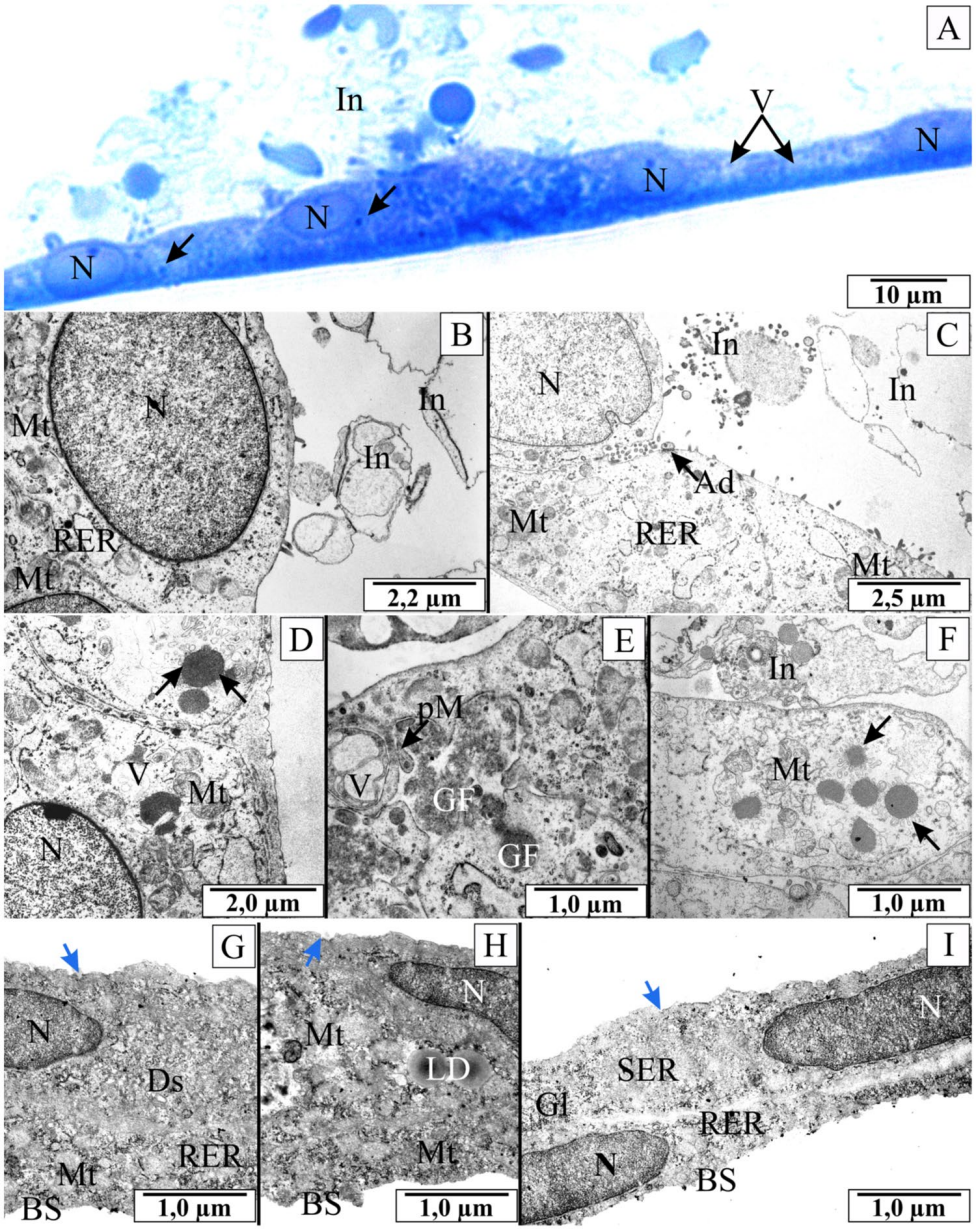
The histological and ultrastructural structure of NRK-52E cells cultured with *B. microti*. (**A**) semithin cross-section of NRK-52E cells stained with methylene blue. Arrows indicate granules inside the epithelial cells. (**B–F**) electronmicrographs of NRK-52E cells. In some cells, vacuolisation inside the cytoplasm was noted. The structure of the cytoplasm was heterogeneous. Large amounts of erythrocytes shadow with granules were visible above the cell free surfaces. (**G–I**) electronmicrographs of NRK-52E cells cultured with blood collected from *Babesia*-negative rat’s. Arrows in figure (**D**,**F**) indicate oval or round structures with high osmophilicity, surrounded by a ring of small elongated elements. Blue arrows in figures (**G,H**,**I**) endocytic membrane pits. Ad—arrow in figure (**C**) indicates adhesion between *B. microti* and renal plasmalemma, *BS* basal side of cells, *Ds* dictyosome, *GF* granular-fibrous material, *Gl* glycogen, *In* *inoculum* with *B. microti*, *LD* lipid droplets, *Mt* mitochondria, *N* nucleus, *pM* arrow in figure (**E**) indicates pyriform merozoite, *RER* rough endoplasmic reticulum, *SER* smooth endoplasmic reticulum, *V* vacuoles.

The microscopic analysis of semi-thin sections through the culture layer of renal epithelial cells infected with *B. microti* showed the cell layer's continuity. In some cells, vacuolisation within the cytoplasm was noted. The structure of the cytoplasm was clearly heterogeneous. Large amounts of erythrocytes shadow with granules were visible above the cells' free surface. Granules of a similar shape and size were found inside the epithelial cells (Fig. 3A). The figures showed cross-sections through the cell monolayer, so the inclusions visible next to the structural elements of the cells were also inside them. During the analysis of the electronmicrographs, it was possible to determine whether the parasites were inside the cells or between them.

Electronmicrographs of NRK-52E cells infected with *B. microti* revealed the inoculum elements above the cells' free surface (Fig. 3B,C,F). The piroplasm adhesion was evident to cells' external surface in cultures supplemented with invaded rats' blood collected. Structures resembling *B. microti* in shape and size directly adhered to the epithelial cell membrane. An electron-dense layer was visualised at the site of adhesion, suggesting close contact between protozoan cell membranes and renal epithelial cells (Fig. 3C).

The elements with doubled cell membrane resembling *B. microti* were observed in numerous cross-sections through the infected NRK-52E. These structures were observed between cells and also in the cytoplasm (Fig. 3E). The presence of granular-fibrous material was found in these places, which indicated local damage to the cells and the ongoing necrosis process. The cytoplasm structure showed symptoms of relaxation and vacuolisation in many areas. Dilated endoplasmic reticulum was also observed in many cells. The mitochondria were enlarged and distorted. The mitochondrial matrix was characterised by heterogeneous osmophilicity. The number of mitochondrial cristae was clearly reduced.

One of the most significant features observed in NRK-52E cells after contact with *B. microti* was the presence of oval or round structures with high osmophilicity, surrounded by a ring of small elongated elements, often connected (Fig. 3D,F). Moreover, the groups of oval or pear-shaped inclusions were visible in many cytoplasm areas.

The control NRK-52E epithelial cells were characterised by high cytoplasmic osmophilicity. Organelles of these cells showed the correct structure. Mitochondria were characterised mainly by an orthodox structure with low amounts of mitochondrial cristae, low matrix osmophilicity, and narrow intermembrane spaces. The endoplasmic reticulum was quite extensive, formed from normal, non-widened tubules. Both rough and smooth endoplasmic reticulum were visible in the sections. Besides, many polyribosomes were visible in the cytoplasm. In many fields of view, the reserve materials, i.e. lipid drops and α-glycogen was observed (Fig. 3G–I).

### Histological and ultrastructural analysis of the effect of *B. microti* on rats’ kidney

**Figure 4:**
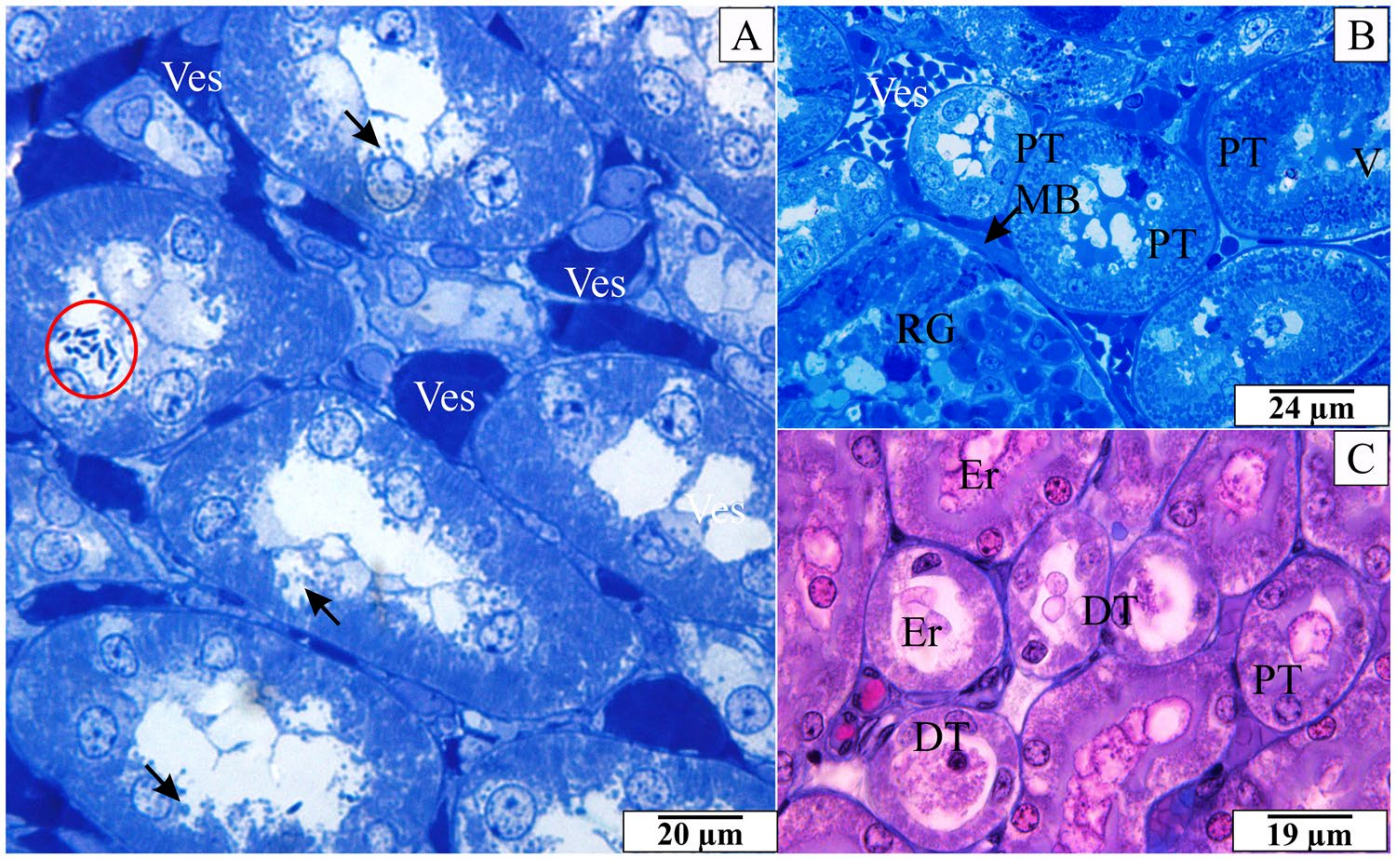
The histological structure of the rats’ renal medulla invaded *B. microti*. (**A,B**) semithin cross-sections through the renal medulla stained with methylene blue. Arrows in figures (**A**) indicate various forms of epithelial cells’ damage. Red circle in figure (**A**) indicate granular or pyriform structures in the proximal tubule. (**C**) cross-section through the renal medulla stained by the AZAN trichrome method. *DT* distal tubules, *Er* erythrocytes, *MB* basal membrane (thickness), *PT* proximal tubules, *RG* renal glomerulus, *V* vacuoles, *Ves* capillary.

In cross-sections through the rats’ kidney, similar destructive changes in the epithelium of renal tubules were observed. During histological examinations, the vacuolization process within the epithelial cells of the proximal tubules was observed. Figure 4B shows such structures surrounded by a cell membrane and filled with fluid. Different intensities of the cytoplasm coloration were observed. Many granular structures were found in many proximal convoluted tubules in the basal part or around the cell nucleus (Fig. 4A). The epithelium of proximal convoluted tubules was also discontinued in some sections. Deposits in the lumen of the tubules and erythrocytes were observed in all segments, i.e., proximal convoluted tubules, loops of Henle, distal convoluted tubules and connecting tubules (Fig. 4A–C). In the presented figures, the described inclusions were located in the nucleus area under the cell membrane. Additional confirmation of the intracellular presence of *B. microti* was the ultrastructural analysis. This allowed distinguishing *Babesia* merozoites originating in damaged erythrocytes from those in the kidney cells. Embolism in the vessels and an accumulation of the erythrocytes were visible in cross-sections. Clusters of erythrocytes causing embolisms were visible in many sections through the renal medulla (Fig. 4A,B). A very close adherence of erythrocytes and thrombocytes to the vascular endothelium was also observed.

**Figure 5:**
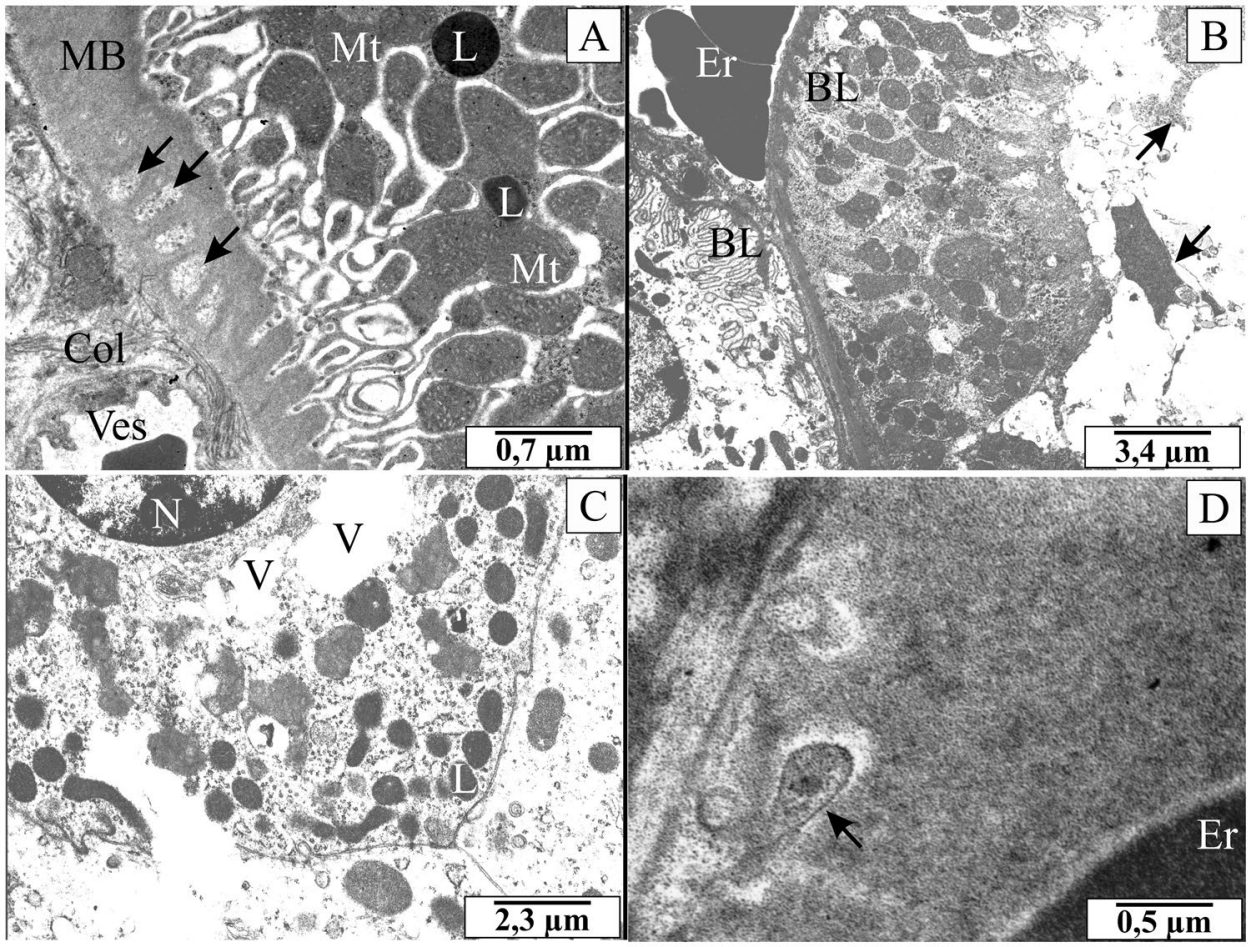
The electronmicrographs showing the rats’ renal medulla invaded *B. microti*. (**A**) the ultrastructure of the basal membrane with inclusions (arrows). (**B,C**) the ultrastructure of the renal epithelial cells with characteristic vacuolisation and damages. Arrows indicate cellular debris, including erythrocyte fragments. (**D**) merozoite of *B. microti* in the basal membrane (arrow). *BL* basal labyrinth, *Col* collagen, *Er* erythrocytes, *L* lysosomes, *MB* basal membrane, *Mt* mitochondria, *N* nucleus, *V* vacuoles, *Ves* capillary.

The ultrastructural observation showed *B. microti* merozoites located in the endothelium of blood vessels and connective tissue between renal tubules (Fig. 5A). Significantly, the localisation of protozoa in endothelial cells was associated with changes in the structure of the cytoplasm of these cells, loosening of connective tissue and changes in the basal membrane of distal convoluted tubules. The presence of oval or round osmophilic elements in some cells of renal tubules was also noted (Fig. 5D). These elements were similar in structure (size, oval or pear-shaped, with a visible cell nucleus and double cell membrane) to the intraplasma inclusions observed in NRK-52E cells cultured in vitro.

The ultrastructure of the renal epithelial cells has shown characteristic vacuolisation, damages and cellular debris, including erythrocyte fragments (Fig. 5B,C). Large amounts of deposits were observed within the thickened basal membrane of the renal tubules. There were strands of connective tissue with collagen fibres (Fig. 5A). An increased amount of collagen seems to be related to enlargement of the space between the blood vessels and the renal tubules and changes in the basal membrane of the renal tubules. In these areas, *B. microti* merozoites were very often visible.

## Discussion

Due to the intense blood flow through the kidneys, these organs are susceptible to pathogenetic blood factors. As a result, the most common acute babesiosis complications include damage to the kidneys, liver and spleen^17–21^. Microscopic studies indicate changes in the proximal tubules, while in the latter stages of the disease, there may be mesangial hypertrophy and glomerulonephritis^23–25^.

Studies on changes in enzymes' activity in the blood of animals suffering from babesiosis confirmed renal dysfunction. Slade et al.^23^ reported a case of a Labrador retriever with nephropathy due to *B. gibsoni* infection. Histopathological examinations of the kidney biopsy showed a mild mesangium overgrowth in the glomeruli, inside which there were strongly osmophilic deposits. Necrotic cells or apoptotic bodies were present in both the renal glomeruli and renal tubules. The discussed morphological and ultrastructural observations of rats' kidneys showed signs of secondary mesangial and membranous-proliferative glomerulonephritis. Inflammatory changes in the renal bodies and necrosis of many renal tubules cells indicated impairment of the kidneys' physiological functions. Similar results were presented by Annable and Ward^26^, who examined the kidneys of rats infected with *B. rodhani*. Histological analyses were performed at different infection stages (from 3rd day to 4th week after infection). The authors showed pathological changes in the renal glomeruli, which appeared as early as 4th day of parasitemia. Studies using immunofluorescence methods have also demonstrated IgG deposits and the complement component C3 in the glomerular capillaries. Tubular cell swelling was due to the presence of intracellular hematin deposits.Blum et al.^27^ presented a patient's case after splenectomy with hemolytic anaemia and acute renal failure symptoms. In the blood smear, the patient was diagnosed with babesiosis, with approximately 30% parasitemia. The kidney biopsy revealed acute tubular damage and kidney inflammation. The authors of the cited report did not provide the exact causes of acute tubular damage and kidney inflammation. The excretory system's complications in a 56-year-old man in the course of babesiosis caused by *B. microti* were also described by Luciano et al.^25^. In this case, studies by light microscopy showed acute tubular damage, oedema of endothelial cells and interstitial infiltration of macrophages, lymphocytes and plasma cells in biopsy material. Macrophages with fragments of erythrocytes appeared in the lumen of the renal tubules. There were no significant changes in renal glomeruli.

In our study numerous destructive changes in renal tubule cells were visible in cross-sections through the renal cortex and medulla. The degeneration was mainly characterised by vacuolisation changes in epithelial cells, observed in preparations analysed by light and transmission electron microscopy. Some of the degenerative changes were so large that they disrupted the renal tubular epithelium's continuity. Deposits, cell fragments and erythrocytes in the lumen of the tubules were present in many fields of observation. These analyses confirmed the powerful influence of the invasion on the structure of the renal tubules. Krawitz et al.^28^ confirmed the occurrence of complications from the excretory system in the course of human babesiosis.

In histological and ultrastructural studies of the dogs' kidney with babesiosis caused by *B. canis*, Máthé et al.^29^ showed degenerative changes mainly in the proximal tubules. The destruction included cell vacuolisation, detachment of tubular epithelial cells from the basal membrane, and in some areas, necrosis of cells and even entire tubules. In the lumen of the tubules, the presence of necrotic debris and deposits of haemoglobin was observed. Authors recognised hypoxia as the cause of renal tubular necrosis, which was associated with anaemia in the course of babesiosis due to the damage of erythrocytes. They also noted hypoxia due to vasoconstriction. In our studies, adherence of blood cells to the vascular endothelium was demonstrated in many sections. Sequestration was present in cross-sections through the renal cortex and medulla, observed by light microscopy. Emboli in intertubular vessels and accumulation of erythrocytes were visible in fields of view. Further ultrastructural analyses of the kidney revealed the close adherence of erythrocytes and thrombocytes to the vascular endothelium. There were no such changes in the renal glomeruli.

In 1977, Hussein^30^ described changes in kidneys of mice infected with *B. hylomysci*. The microscopic analyses by light microscopy showed hyaline droplets, occasional intracellular vacuoles in the proximal tubules, and various tubular necrosis stages. In the lumen of the tubules, there were glassy, granular and cellular cylinders. The toxic effect of the haemoglobin released from erythrocytes during lysis was responsible for the renal tubules' damage, as hemosiderin deposits accumulated in the epithelial cells of the proximal tubules. In our studies, damage to the renal tubular cells, such as vacuolation and discontinuity of the epithelial layer, was also observed.

In vivo studies showed vacuoles in the renal epithelium, which was a manifestation of the necrosis process. Similar changes were observed in material collected from in vitro cultures. Reduced osmophilicity of cytoplasm indicated dilution and altered protein profile due to biochemical degradation. Moreover, these cells were characterised by an increased number of primary and secondary lysosomes involved in these processes. The presence of residual bodies and granular-fibrous material within the cytoplasm of NRK-52E cells was a symptom of autophagy in cells, related to the destructive processes, which various mechanisms can cause. First, the influence of protozoan metabolites may be significant. In addition, the binding of *B. microti* to receptors allows piroplasm to exist inside infected cells. The parasite can trigger a cascade of intracellular immune responses that can provoke the infected cells to death^31^.

Changes in the cells of NRK-52E also focused on other organelles. The observed mitochondria were characterised by oedema, reduced number of mitochondrial cristae and heterogeneous matrix osmophilicity. Differences in mitochondrial matrix osmophilicity (significantly its increase) indicate decreased ATP/ADP ratio, which results in permanent cellular hypoxia. This process is associated with increased lysosome activity and autophagy of abnormal mitochondria and other areas of the cytoplasm. Such damage of organelles is described as a symptom of cell necrosis^32,33^. Lysosomal enzymes begin the digestion of cellular components, which leads to their disintegration and the release of contents^15^.

Inside NRK-52E cells, the presence of peroxisomes was also demonstrated. These structures are involved in the fight against oxidative stress. As a result of the protozoa invasion, the production of reactive oxygen species increases. It is believed that during the infection, free radicals play an essential role in tissue damage through DNA degradation and lipid peroxidation^34^.

It has been shown in animal models that erythrocytes invaded with *B. bovis* induced an acute inflammatory response and activated the coagulation system, leading to increased adhesion of blood cells to capillaries^35^. Similar changes were observed in our studies.

Cytoadhesion, protozoa adherence to blood cells, adhesion of blood cells to each other, and endothelial cells result in disturbance of hemodynamic processes in the kidney's microcirculation. Disturbances in blood microflow may contribute to the activation of the coagulation cascade. These processes, leading to emboli formation, cause hypoxia of tissues and organs, which may cause necrotic changes. In our study, ultrastructural observations showed that *B. microti* merozoites were localized in the endothelium of blood vessels and the connective tissue between renal tubules. Adhesion of protozoa to blood cells, adhesion of blood cells to each other, and endothelial cells can induce an acute inflammatory response and activate the coagulation system, leading to increased adhesion of blood cells to capillaries. Thus, *B. microti* can directly induce inflammation, but inflammatory factors are indirectly responsible for the cascade of embolic processes.

The presented results indicate many similar changes in cells cultured in vitro and tissues collected from rats infected by *B. microti*. The observations show that, to a certain extent, *B. microti* exerts pathogenic effects directly on cells and tissues. The secondary effects of animal infection may potentiate or inhibit parasitemia, depending on the organism's immune condition. Nevertheless, the effects of *B. microti* on cells in vivo and in vitro are similar in many aspects.

In summary, both in vivo* and *in vitro, there is a chemotactic tendency for *B. microti* to adhere to the renal tubules' epithelium. In addition, there is a possibility of piroplasms entering the renal epithelial cells and their proliferation within the cytoplasm, which results in necrotic changes. In vivo, *B. microti* is the cause of the emboli formation in the rat kidney. There are also indications that protozoa disrupt the blood-renal epithelium barrier. The results presented in this study show that *Babesia microti* can penetrate the epithelial cells of the renal tubules. Nevertheless, we must exercise some caution in specifying such an opinion and recognizing it as universal. This caution is due to two aspects. First, the in vitro behavior of parasitic *B. microti* can be significantly different from the in vivo situation. There are no immune mechanisms in cell culture that can prevent this in organisms with a properly functioning immune system. On the other hand, the microscopic documentation confirms that in in vivo conditions, penetration of rat kidney cells and endothelial cells also occurred. Moreover, this penetration led to marked degenerative changes in epithelial cells. However, rodents (including rats) are naturally reservoir species for *B. microti*. It may cause *B. microti* to behave differently in casual hosts such as humans, which may be significant from a medical point of view.

## Methods

### In vivo studies

In the in vivo study, males of Wistar rats (12 weeks old, weighing 320–350 g) were used and randomly assigned to the study group (N = 10) and the control group (N = 3). The animals were obtained from Department for Experimental Medicine (Katowice, Poland). The animals were housed in standardized sterile conditions (temperature: 20–22 °C, humidity: 50–60%, lighting cycles: 12 h light and 12 h darkness). Breeding was conducted in cages with pine sawdust which was autoclaved before use. These conditions prevented the contamination of the animals used in the experiment with other pathogens. The animals had unlimited access to water and fodder.

The study rats' (all 10 individuals from the test group) inoculation procedure consisted of injection intraperitoneally 0.5 ml of blood from the second passage of rats (2 individuals), collected on the 21st day after infection (parasitemia about 20%). A reference strain culture of *B. microti* (Franca) Reichenow ATCC 30221 (ATCC, Manassas, VA, USA) was used for the primary infection. Parasitemia control was performed in all rats at weekly intervals. The control group was injected with 0.5 ml of saline. After 21 days from inoculation, i.e. with the observed increase of parasitemia, the rats were anaesthetised by inhalation of isoflurane (in accordance with American Veterinary Medical Association Guidelines for the Euthanasia of Animals) and then blood samples and kidney were collected. The tissues were fixed in Bouin's solution (saturated picric acid solution, neutral buffered formalin solution 40% pure p.a., and glacial acetic acid, mixed in v/v/v ratio 15:5:1 immediately before use)^36^ and Karnovski's solution (2.5% buffered paraformaldehyde solution and 2.5% buffered glutaraldehyde solution in v/v ratio 1:1)^37^, respectively, to histological and ultrastructural studies. The blood was used to make smears used parasitemia control. The evaluation of parasitemia was performed according to the recommendations attached to the specifications of the used *B. microti* reference strain. 500 successively encountered erythrocytes were observed under the light microscope. During the analyzes, the percentage of blood cells with the presence of inclusions was assessed.

To confirm that of the monolayer cells cultured in vitro contained *B. microti*, the rats (2 individuals) were inoculated by an intraperitoneal injection of 0.5 ml epithelial cell monolayer (rinsed with sterile saline), which had previously been cultured in vitro with blood infected with *B. microti*. After 7 days, the presence of parasitic merozoites in the blood was checked.

### In vitro studies

In the in vitro studies, rats’ kidney epithelial cells (NRK-52E; ECACC 87,012,902; Sigma-Aldrich, St. Louis, MO, USA) were cultured in Dulbecco’s Modified Eagle’s Medium (DMEM) supplemented with 2 mM L-glutamine, 5% fetal bovine serum and 1% non-essential amino acids. Cultures were grown in sterile 12-well Nunclon surface plates (Nunc, Wiesbaden, Germany)—10 wells were used for *B. microti* infection (2 biological replicates from second passage rats and 5 technical replicates from each of 2 rats), 2 wells were used as controls. After reaching confluence, 0.5 ml of rats blood with the confirmed presence of the *B. microti* was added to the culture (approximately 20% parasitemia, the blood of 2 rats from second passage). The same blood was used in in vivo studies. Control cells were NRK-52E line grown in the above media with the addition of control rats’ blood. Cultures were performed at 37 °C for 48 h in a 5% CO_2_ atmosphere. After this time, the cultures were rinsed with DMEM and fixed in 2.5% paraformaldehyde in phosphate buffer.

### Parasitemia control

May-Grünwald-Giemsa staining (Aqua-Med ZPAM, Cracow, Poland) was performed to confirm the presence of *B. microti* in rats’ blood smears fixed in methyl alcohol. The procedure was in accordance with the manufacturer's instructions. Preparations were observed using Olympus BX60 microscope. The presence of protozoan DNA was also confirmed in blood smear, epithelial cells and rats’ kidney using the fluorescent in situ hybridization (FISH). Histology FISH AccessoryKit (DAKO, Carpinteria, CA, USA) was used to detect *B. microti* genetic material. The procedure of fluorescent staining was following the manufacturer's instructions. The probe sequence was as follows: 5’-fluorescein-GCCACGCGAAAACGCGCCTCGA-fluorescein-3’ (Metabion, Planegg, Germany)^38^. The probe was complementary to the fragment of *B. microti* 18S rRNA gene. The preparations were analysed using the Olympus BX60 epifluorescence microscope, in which the source of the excitation radiation was a xenon lamp with a power of 150 W.

Blood collected from the animals was centrifuged at low speed, and blood cells were fixed in 2.5% paraformaldehyde solution at 4 °C and analysed by transmission electron microscopy (TEM). The material preparation steps followed standard histological methodology.

### Histological and ultrastructural studies

The fixed material from in vitro culture and fixed in Karnovski's solution fragments of rats’ kidney were rinsed in phosphate buffer to remove the fixative solution and embedded in epoxy resin (Poly/Bed^®^812 Embedding Media/DMP-30 Kit, Polyscience, Inc., Warrington, PA, USA) according to the standard methodology used in histological studies^39^. The material obtained from in vitro culture was dehydrated in ethanol-acetone series and embedded in epoxy resin directly on the culture plates.

The obtained epone blocks were cut with a Leica Ultracut UCT ultramicrotome. Semi-thin sections were stained with 1% methylene blue solution at high temperature. For TEM analyses, the ultra-thin sections were contrasted with uranyl acetate and lead citrate. Observations were made using a Hitachi H500 Transmission Electron Microscope with an accelerating voltage of 75 kV.

The tissues for histological examination were fixed in Bouin's solution, rinsed in ethanol series and embedded in paraffin blocks. Paraffin sections were stained using AZAN trichrome method (stain used: 0.1 g azocarmine G/100 ml distilled water, 2 g orange G/100 ml distilled water and 0.5 g aniline blue/100 ml distilled water). Staining was performed according to standard histological methodology. Histological preparations were observed using Olympus BX60 microscope.

### Statements

The authors declare that all in vivo experiments were approved by the Local Ethics Committee for Animal Experiments in Katowice, Poland (approval no. 32/2011).

The authors also declare that all methods used in the studies were carried out in accordance with ethical guidelines and legal regulations. All procedures were carried out according to the protocol based on the European Convention for the Protection of Vertebrate Animals used for Experimental and Other Scientific Purposes, approved by local ethical committee.

The authors confirm that the study was carried out in compliance with the ARRIVE guidelines.
